# Results of Multilevel Containment Measures to Better Protect Lung Cancer Patients From COVID-19: The IEO Model

**DOI:** 10.3389/fonc.2020.00665

**Published:** 2020-04-22

**Authors:** Filippo de Marinis, Ilaria Attili, Stefania Morganti, Valeria Stati, Gianluca Spitaleri, Letizia Gianoncelli, Ester Del Signore, Chiara Catania, Cristiano Rampinelli, Emanuela Omodeo Salè, Lorenzo Spaggiari, Fabrizio Mastrilli, Antonio Passaro

**Affiliations:** ^1^Division of Thoracic Oncology, IEO, European Institute of Oncology IRCCS, Milan, Italy; ^2^Division of Early Drug Development for Innovative Therapies, IEO European Institute of Oncology IRCCS, Milan, Italy; ^3^Department of Oncology and Hemato-Oncology, University of Milan, Milan, Italy; ^4^Department of Radiology, IRCCS European Institute of Oncology, Milan, Italy; ^5^Department of Pharmacy, IRCCS European Institute of Oncology, Milan, Italy; ^6^Division of Thoracic Surgery, IEO, European Institute of Oncology, IRCCS, Milan, Italy; ^7^Medical Administration, CMO, IEO, European Institute of Oncology, IRCCS, Milan, Italy

**Keywords:** COVID-19, coronavirus, cancer, lung cancer, containment measures

## Abstract

A novel coronavirus causing severe acute respiratory syndrome (SARS), named SARS-CoV-2, was identified at the end of 2019. The spread of coronavirus disease 2019 (COVID-19) has progressively expanded from China, involving several countries throughout the world, leading to the classification of the disease as a pandemic by the World Health Organization (WHO). According to published reports, COVID-19 severity and mortality are higher in elderly patients and those with active comorbidities. In particular, lung cancer patients were reported to be at high risk of pulmonary complications related to SARS-CoV2 infection. Therefore, the management of cancer care during the COVID-19 pandemic is a crucial issue, to which national and international oncology organizations have replied with recommendations concerning patients receiving anticancer treatments, delaying follow-up visits and limiting caregiver admission to the hospitals. In this historical moment, medical oncologists are required to consider the possibility to delay active treatment administration based on a case-by-case risk/benefit evaluation. Potential risks associated with COVID-19 infection should be considered, considering tumor histology and natural course, disease setting, clinical conditions, and disease burden, together with the expected benefit, toxicities (e.g., myelosuppression or interstitial lung disease), and response obtained from the planned or ongoing treatment. In this study, we report the results of proactive measures including social media, telemedicine, and telephone triage for screening patients with lung cancer during the COVID-19 outbreak in the European Institute of Oncology (Milan, Italy). Proactive management and containment measures, applied in a structured and daily way, has significantly aided the identification of advance patients with suspected symptoms related to COVID-19, limiting their admission to our cancer center; we have thus been more able to protect other patients from possible contamination and at the same time guarantee to the suspected patients the immediate treatment and evaluation in referral hospitals for COVID-19.

## Introduction

On March 11th, 2020, the World Health Organization declared the COVID-19 outbreak as a pandemic. By April 5th, 2020, more than 1.21 million of cases with more than 65,600 deaths have been reported worldwide, with a continuously increasing trend in the number of infections and fatal cases (1).

In March 2020, following China, Europe became the new epicenter of the pandemic. Italy is one of the worst-affected European countries, and Lombardy is its most severely hit region, with more than 120,000 cases and more than 15,000 deaths registered since January 31st (1, 2).

Few data are available about the prevalence of cancer history in patients with a COVID-19 infection (3). The Chinese Centre for Disease Control and Prevention (CDCC) reported a history of cancer in 107 patients among 44,672 confirmed COVID-19 cases (prevalence 0.5%), with six deaths (1.5% of total deaths) and a case fatality rate of 6.5% (4). Liang et al. registered a similar prevalence of cancer history (1%, 18 patients) among 1,590 patients with a COVID-19 infection. In this analysis, cancer history represented the highest risk factor for severe adverse events after adjusting for age, sex, and comorbidities (3).

European data suggest a significantly higher prevalence of active cancer among patients affected by COVID-19. In Italy, a retrospective analysis of medical records of 710 patients dead with COVID-19 infection found a diagnosis of active cancer in 155 of them (17.3%) (3).

Data about the prevalence of COVID-19 infection among patients with cancer are sparse. The Zhongnan Hospital of Wuhan University reviewed health records from 1,524 patients with cancer, reporting a diagnosis of COVID-19 infection in 12 patients (0.79%) (5). Based on these data, the infection rate among patients with cancer seems to be higher than in the general Chinese population (0.37%; 41,152 of 11,081,000 cases). To our knowledge, no data explicitly addressing the spread of COVID infection in patients with cancer outside China have been published.

Patients with lung cancer might have an even higher risk of COVID-19 infection and a worse prognosis (6). In addition to a general predisposing immunosuppressive status induced by anticancer therapies, several factors that have been associated with an unfavorable outcome from.

COVID-19 infection have been frequently reported in patients with lung cancer, such as male sex, cardiovascular comorbidities, and chronic respiratory diseases (4, 7).

Among the 12 cancer patients with COVID infection reported by Yu et al., seven had lung cancer, and two of them died after experiencing severe COVID (5). Lung cancer was the most frequent histology also in the analyses published by Liang et al. (3) (five out of 18 patients) and Zhang et al. (8) (seven out of 28 patients).

Following the initial spread of COVID-19 in Italy, a lockdown was imposed on February 23rd by the Italian government; it covered 10 municipalities of the province of Lodi in Lombardy, 30 km from Milan. On March 11th, the lockdown was extended to all of Italy.

Since late February 2020, cancer care in Italy has needed to be re-organized, but specific guidelines concerning the management of patients with cancer have been lacking.

On March 10th, the Italian National Health System firstly released a recommendation paper regarding the treatment of cancer patients during COVID-19 pandemic; this was followed by a more detailed guideline document published on March 13th by the Italian Association of Medical Oncology (AIOM) (9). On March 20th, the National Comprehensive Cancer Network (NCCN) also published specific recommendations for the management of cancer patients in endemic areas (10), followed by the European Society of Medical Oncology (ESMO) on March 21 (11).

The European Institute of Oncology (IEO) is a Comprehensive Cancer Center located in Milan, Lombardy. Since the beginning of the COVID-19 spread, we have applied a protocol of proactive management of cancer patients based on telephone triage, delaying of selected scheduled therapies and restricted access to IEO Institution. This analysis has aimed to prove that such proactive management allowed for the minimization of contagion among patients with lung cancer through the maximization of preventive measures.

## Materials and Methods

Since the COVID-19 outbreak in Italy, we early on designed internal guidelines to allow our patients to continue treatment for their cancer and reduce COVID-19-related risks safely.

Here the internal evaluation steps for all patients with lung cancer admitted to our cancer center:

1. Before the Institute admission: all patients received emails periodically and recommendations to follow in order to protect themselves from contagion to avoid spreading the infection; this included essential information to improve hygiene and personal care.
2. *Day 1 of each planned visit or treatment:* all patients were screened by telephone triage at day 1 of each clinical visit for various symptoms: cough, sore throat, headache, dyspnea, fever, oxygen desaturation, chest tightness, myalgia, diarrhea, nausea/vomiting, anosmia, and dysgeusia.
3. *Day of visit/treatment:* clinical triage at the cancer center admission, with clinical evaluation for respiratory tract symptoms and fever check.
4. Patients with symptoms or a chest CT-scan suggestive of interstitial lung disease (ILD) underwent a nasopharyngeal swab for SARS-COV-2 identification.

### Timeline of Activation of Containment Measures Implemented by Our Division of Thoracic Oncology

*From February 24th 2020:* A Division meeting was scheduled twice a week, and it was aimed at discussing for treatment delays of patients scheduled for the subsequent week.

*From March 2nd 2020:* A telephone triage was introduced for all patients, checking for suspected symptoms and personal contacts with people positive or suspected for COVID-19 infection. Dedicated counseling was also implemented to stress to patients the importance of the measures recommended by the Italian Ministry of Health to avoid the virus spreading, i.e., to wear a surgical mask and to maintain a safe interpersonal distance of at least 1.5 m.

- *From March 9th 2020:* Patients' temperature was checked before hospital admission, according to Chief Medical Officer (CMO) guidelines
- *From March 16th 2020:* Access to IEO was forbidden to all people except for patients and employees by the Chief Medical Officer. Visiting time for inpatients was limited to 1 h, and only caregivers of non-self-sufficient outpatients were admitted.

Since patients with lung cancer have a higher risk of developing significant COVID-19-related complications due to both the disease itself and cancer treatment, more specific internal guidelines were outlined by our Division of Thoracic Oncology.

### In-Patient Management Guidelines Adopted by the Division of Thoracic Oncology

Patient evaluation of the risk/benefit ratio for delaying anticancer treatment based on several factors:

1. stage and histology
2. age
3. ECOG Performance Status (PS)
4. treatment type [chemotherapy, immunotherapy, tyrosine kinase inhibitors (TKI)]
5. comorbidities
6. history of recent pneumonitis

· Visit/treatment delaying for patients with recent onset of tract respiratory symptoms or fever.
· Delaying of CT scan/other imaging procedures planned to evaluate treatment response in asymptomatic patients.
· Delivery of oral cancer treatments to authorized pharmacies near to patients' domicile for patients living outside the Lombardy region; all frail patients; and selected clinical trial drugs according to Sponsor indication and authorization.

On March 12th, the Italian Medicine Agency (AIFA) officially authorized local delivery of drugs. Dispensing or delivery of multiple treatment cycles of oral drugs, if feasible, was based on supply availability. For frail patients, drug packs were given to anauthorized caregiver in order to minimize their exposure risk.

· Replacement of scheduled follow-up visits with email or phone call and telematics evaluation of CT scan imaging in the absence of urgent medical needs. Telemedicine evaluation was adopted for follow-up visits and the evaluation and treatment of suspected side effects.
· Avoid delaying of curative treatment or treatment for highly progressive/symptomatic tumors.

We therefore decided to start or continue several treatments:

- Adjuvant/neoadjuvant therapies
- Chemo-radiotherapy for unresectable stage III diseases
- Commencing first-line therapies (chemotherapy, immunotherapy, and tyrosine kinase inhibitors) for metastatic disease
- Chemotherapy for high-grade tumors (small cell lung cancer or large cell neuroendocrine tumors) without any interruption in the absence of suspected symptoms
- Clinical trial treatments.

For patients living far from Milan, we also considered a referral to Cancer Centers nearer to the patients' homes.

We reviewed medical records of all patients with a scheduled visit at the Division of Thoracic Oncology, European Institute of Oncology, from March 2nd 2020 to April 3rd 2020. Patients were categorized according to admission type, and personal details, including age, sex, and region of residence, and clinical data were collected. The adopted proactive management for each patient was recorded.

### Endpoints

The primary endpoint of this study is to identify the feasibility and efficacy of early adoption of proactive management measures in terms of the rate of COVID-19 diagnosis, hospitalization, death among our lung cancer patients, rate of delayed or discontinued treatments, rate of delivered drugs, and the rate of avoided patients' accesses.

Secondary endpoints include the evaluation of the clinical features of managed patients and the compliance of patients and their family to medical oncologists' recommendations in terms of the rate of patients refusing the proposed management.

Variables were presented by using the median value for continuous variables and percentages (numbers) for categorical variables, and their relationship with the adopted measures was assessed using the Mann–Whitney test and the chi-squared test as appropriate.

All statistical tests used a two-sided 5% significance level, and association measures were provided with their 95% confidence interval. Statistical analyses were performed using RStudio (RStudio: Integrated Development for R. RStudio, Inc., Boston, MA).

## Results

Overall, 477 accesses at IEO Division of Thoracic Oncology were scheduled in the considered 5-week timeframe for a total of 325 patients included in our analyses. The median age was 67 years (range 23–89; interquartile region Q1–Q3, 59–73 years), and male to female ratio was 1:1. The majority of patients (59.7%) were living in the Lombardy outbreak region, while 20 out of the 131 patients coming from outside Lombardy were living in other areas of COVID-19 outbreak. Clinical features of the overall patient population are summarized in Table 1.

**Table 1:** Clinical characteristics of study population.

**Total**		***n* = 325**
Age	Median (Q1-Q3)	67 (59–73)
	<65 yrs	132 (40.6%)
	65-75 yrs	135 (41.5%)
	≥ 75 yrs	58 (17.9%)
Gender	Male	164 (50.5%)
	Female	161 (49.5%)
Region of residence	Lombardia	194 (59.7%)
	Out-Lombardia	131 (40.3%)
	Red zone	20 (15.3%)
	No red zone	111 (84.7%)
Smoking	Yes	62 (19.1%)
	No	105 (32.3%)
	Former	158 (48.6%)
ECOG PS	0	86 (26.5%)
	1	210 (64.6%)
	2	25 (7.7%)
	3	4 (1.2%)
Disease setting	Early stage (I-II)	14 (4.3%)
	Locally advanced (III)	32 (9.8%)
	Metastatic (IV)	279 (85.9%)
Histology	NSCLC	300 (92.3%)
	Non-squamous	278 (92.7%)
	Squamous	22 (7.3%)
	SCLC	20 (6.2%)
	Other	5 (1.5%)
Access type	First-access visit	30 (9.2%)
	Follow-up visit	16 (4.9%)
	Cancer treatment	279 (85.9%)
	CHT	74 (26.5%)
	IO	51 (18.3%)
	CHT-IO	6 (2.1%)
	TKI	99 (35.5%)
	Clinical trial	49 (17.6%)
Steroid use	No	179 (55.1%)
	Yes	146 (44.9%)
	Prophylaxis	92 (63%)
	Chronic	54 (37%)
Recent (<6 months)	No	308 (94.7%)
pneumonitis	Yes	17 (5.2%)

*CHT, chemotherapy; IO, immunotherapy; TKI, tyrosine kinase inhibitors*.

Categorizing patients according to access type, we found that:

- 118 (36.3%) were scheduled for standard intravenous (IV) cancer treatment administration
- 112 (34.5%) were planned for receiving oral anticancer treatments
- 49 (15.1%) patients were in clinical trials
- 16 (4.9%) follow-up visits
- 30 (9.2%) first-access visits.

A total of 325 (100%) patients received triage phone call according to internal management guidelines. Overall, 149 (45.8%) patients received a recommendation for delaying or canceling the scheduled access. Only seven out of 149 (4.7%) patients refused the proposed management, resulting in 142 (43.7%) of patients with at least one visit cancellation and a total of 174 out of 477 (36.7%) canceled accesses.

Additionally, a delegate was admitted to scheduled visit instead of the patient in 28 cases, resulting in a final count of 170 out of 325 (52.3%) canceled patients' accesses.

Treatment delay was recommended to 62 out of 118 (52.5%) IV treated patients, with a higher frequency for patients in metastatic setting compared to early-stage or locally advanced (odds ratio OR 3.44, 95% CI 1.33–9.78, *p* = 0.017) and for those receiving immunotherapy compounds compared to chemotherapy alone (OR 3.99, 95% CI 1.86–8.88, *p* < 0.001). Most of the delays were adopted in patients with ongoing long-term treatment (OR 4.69 for patients with at least four previous cycles received, 95% CI 2.13–10.82, *p* < 0.001). No correlation was found between treatment delay and ECOG PS (OR 1.49, 95% CI 0.33–8.03 for PS 2 or greater compared to PS 0-1 (*p* = 0.848) and age (*p* = 0.454) in this subgroup.

The oral treatment was delivered to 71 out of 112 patients by territorial delivery (*n* = 47) or shipment to delegate person (*n* = 24). This measure of management was predominantly adopted for patients with ECOG PS ≥ 1 rather than PS0 (*p* = 0.033) and was found to be correlated with age (median age 68.5 vs. 62.5; *p* = 0.011). Two additional patients in this subgroup moved their treatment to cancer centers next to their residency, and three patients had their visit canceled due to disease progression, with an overall patient access reduction of 67.9%.

As for patients in clinical trials or compassionate use of investigational drugs, we found 22.4% treatment delays: seven out of 49 patients with canceled access due to worsening of clinical conditions related to cancer and four patients with delayed treatment. Oral experimental treatments were delivered to 11 patients (22.4%) in accordance with trial sponsors and IEO Pharmacy Unit.

Follow-up visit cancellation was proposed to eight out of 16 patients (50%) upon telematic consultation of required radiological examination, and first-access visits were avoided in two out of 30 (6.7%) patients following referral to residence region or delegate consultation.

During the pandemic timeframe, 148 patients (45.5%) underwent regularly planned thorax CT scan for disease evaluation. Ground-glass opacities (GGO) were detected in 12 cases (8.1%); of them, nine (75%) were receiving immunotherapy or TKI compound. Upon consultation with a radiologist, six cases (50%) were considered suspect for COVID-19 and underwent a swab test: five out of six patients (83%) exhibited a positive swab test for SARS-CoV2 infection.

Overall, nine patients (6.1%) underwent a swab test according to clinical symptoms or radiological findings, resulting in a positivity rate of 66.7% (*n* = 6).

At the data cut-off, 6 (1.8%) of the entire patients' population had confirmed SARS-CoV2 infection (Table 2). Among patients who tested positive for COVID-19, the hospitalization rate was 33.3% (2/6), with only one patient (16.7%) requiring oxygen support, and no deaths occurred.

**Table 2:** Demographic and clinical characteristics of NSCLC COVID 19 patients.

**Patient number**	**1**	**2**	**3**	**4**	**5**	**6**
Sex	Man	Man	Woman	Woman	Man	Woman
Age, years	41	48	59	47	69	62
Smoking status	Current	Former	Never	Former	Former	Never
ECOG PS	1	1	1	0	1	1
Geographical area (region)	Lombardia	Lombardia	Emilia Romagna	Lombardia	Lazio	Lombardia
Co-morbidities	No	No	No	No	Yes	Yes
Histology	Non-squamous	Squamous	Non-squamous	Non-squamous	Non-squamous	Non-squamous
Stage	IV	IV	IV	IV	IV	IV
Clinical Trial	Yes	No	Yes	No	No	No
Treatment regimen	CHT-IO	IO	TKI	IO	IO	CHT
Line arms	1L	1L	2L	1L	2L	1L
No. of doses received	5	93	8	7	38	3
Phone call triage	Yes	Yes	Yes	Yes	Yes	Yes
Dilatation of anticancer treatments	No	Yes	No	Yes	Yes	No
Time between onset COVID 19 diagnosis and last clinical evaluation	11 days	1 day	10 days	26 days	37 days	21 days
Clinical Symptoms	Fever; cought; headache	None	None	Fever; cought; conjunctivitis; dysgeusia	Fever; myalgia	Rhinorrhea; cought
Concomitant treatment	Steroid	Steroid	Steroid	Steroid	Sartan	Steroid
Prior surgery	Yes	Yes	No	Yes	No	Yes
Prior RT	No	Yes	Yes	Yes	Yes	Yes
Contact with confirmed COVID 19 patient	Not certain	Not certain	Not certain	Not certain	Yes	Yes
CT diagnosis	Positive	Positive	Negative	Positive	Positive	Positive
SAR-COV 2 RT-PCR assay	Positive	Positive	Positive	Positive	Positive	Positive
Hospitalization status	Discharged	Not hospitalized	Not hospitalized	Not hospitalized	Inpatient	Not hospitalized
COVID 19 management	Darunavir/ritonavir/Hydroxychloroquine	Self-isolation at home	Hydroxychloroquine	Hydroxychloroquine	CPAP	Self-isolation at home
Survival status	Alive	Alive	Alive	Alive	Alive	Alive

*NSCLC, non-small cell lung cancer; PS, perfprmance status; IO, immunotherapy; CHT, chemotherapy; RT, radiotherapy; TKI, tyrosine kinase inhibitor; CT, computed tomography; RT-PCR, real-time quantitative polymerase chain reaction; SARS-COV-2, severe acute respiratory syndrome coronavirus 2; CPAP, continuous positive airway pressure*.

## Discussion

Coronavirus disease 2019 (COVID-19) has dramatically changed our work as healthcare professionals and our perception of danger, both for ourselves and our patients.

At present, because of its extreme celerity of diffusion, it is difficult to analyze and deeply understand the impact of COVID-19 on a different kind of patient's population. However, data reported by the Chinese Center for Disease Control and Prevention (CDCC) confirmed that patients with multiple comorbidities or cancer showed a higher risk of COVID-19 infection and a worse prognosis (7). Although data are limited, three different retrospective studies report lung carcinoma as the most frequent cancer type, burdened by a worse prognosis (3, 5, 6, 8).

In addition to a general predisposing immunosuppressive status induced by anticancer therapies, several factors that have been associated with an unfavorable outcome from COVID-19 infection are frequently clustered in patients with lung cancer, such as male sex, cardiovascular comorbidities, and chronic respiratory diseases (6).

These reports, associated with awareness of the weakness of cancer patients, have led to the release of different national or international recommendations suggesting how to manage and treat patients affected by different kind of cancers during the COVID-19 epidemic (9, 12). To date, no data are available to confirm whether delaying or avoiding some cycle of cancer treatment will affect patients prognosis, but, of course, we know that in some cases the risk of hospitalization related to SARS-CoV-2 infections could be much more harmful and potentially lethal (13). The Italian National Health System released the first institutional recommendation, specifically addressing patients with cancer on March 10th, followed by AIOM on March 13th (14).

In our clinical practice, from the advent of COVID-19 outbreak, we anticipated the National and Institutional guidelines, and, since February 24th, we have been implementing recommendations and advice to improve the management of lung cancer patients receiving active cancer treatments, e.g., chemotherapy or immunotherapy of tyrosine kinase inhibitors.

Today, although the implementation of these recommendations appears essential for disease management, we have tried to improve the assistance to our patients starting from a few steps of evaluation before the clinic admission, where proactive management and prevention can be incidental and protective for the patient's health.

In our Division of Thoracic Oncology at European Institute of Oncology, located in Lombardy, among the most affected areas worldwide with multiple red zones, we have acted a multilevel strategy using different barrages and containment measures, to inform, educate and create awareness of the risk in our patients and their caregivers.

It is highly recommended to deeply inform cancer patients regarding the SARS-CoV-2 infection and to train them in social distancing, avoiding crowded spaces, especially indoors, and close contact with other people, family members, and caregivers, as well as in improving their personal hygiene, starting from an accurate handwashing habit.

**Figure 1:**
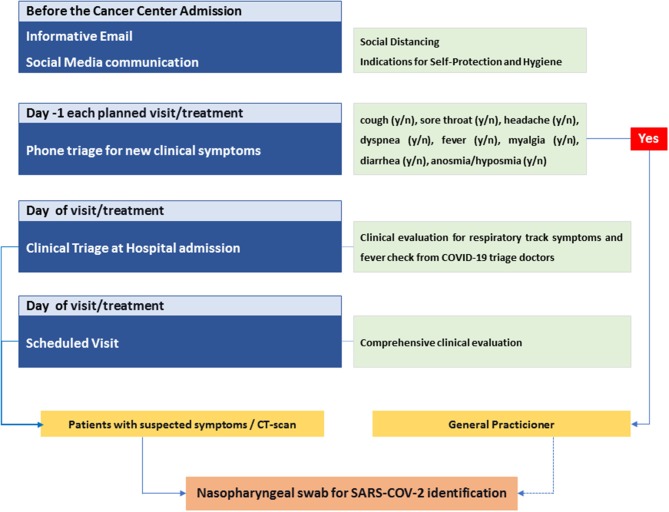
Flow chart of applied multilevel containment measures.

Education of patients and counseling measures appeared crucial to prevent the spread of COVID-19, not only inside our Institution but also outside it, during patients' daily routine. We used two different way of information to reach our patients: email and social media (see Figure 1).

Since the beginning of the COVID-19 outbreak in Italy, all of our patients received a triage phone call one or two times a week, close to the scheduled appointments for treatment or follow-up, to investigate clinical condition, any new respiratory symptoms, or fever. Follow-up visits were converted to telemedicine evaluation by phone or email, and patients scheduled for treatment were evaluated on a case-by-case basis, taking into account overall disease features, prognosis, and clinical condition.

Over about 1 month of phone call triage involving 325 patients, 45.8% of them received the recommendation from our medical staff to delaying or canceling the scheduled access, with only seven (4.7%) of them refusing the proposed management (see Table 3).

**Table 3:** Results of multilevel containment measures for COVID-19.

**Total patient evaluated**		***n* = 325**
SARS-CoV2 testing		9 (6.1%)
	Swab test +	6 (1.8%)
	Deaths	0 (0%)
Modified scheduled visit/treatment		170 (52.3%)
	Delay	142 (43.7%)
	Delegate access	28 (16.5%)
Scheduled treatment		n = 279 (85.8%)
I.V.		118 (42.3%)
	Delayed	62 (52.5%)
Oral		112 (40.1%)
	Access modified	76 (67.9%)
	Delivery	47 (61.8%)
	Delegate access	24 (31.6%)
	Other reasons	5 (6.6%)
Clinical trials		49 (17.6%)
	Access modified	22 (44.8%)
	Delayed	11 (50%)
	Territorial delivery	11 (50%)
Visit		*n* = 46 (14.2%)
Follow-up		16 (34.8%)
	Delayed	8 (50%)
First access		30 (65.2%)
	Delayed	2 (6.7%)

To improve cancer care and reduce potential exposure to SARS-CoV-2 infection, 47 patients received their oral treatment (chemotherapy or tyrosine kinase inhibitors) delivered at home.

For all patients with confirmed treatment, a second step clinical evaluation was performed:

Clinical triage at the admission to the Cancer Care to check for fever and any newly onset respiratory symptom.

A comprehensive evaluation, investigating social activity and clinical symptoms, with focus on fever, cough, dyspnea, or others suggestive symptoms (sore throat, headache, oxygen desaturation, chest tightness, myalgia, diarrhea, nausea/vomiting, anosmia, and dysgeusia).

Such a careful screening of patients before the admission to our Institution was aimed at minimizing the contact between potentially affected and not-affected subjects, including both patients and healthcare workers.

Among the 325 patients evaluated by phone call triage, only nine patients (6.1%) underwent a swab test according to clinical symptoms or suspected radiological findings, with five COVID-19 cases being confirmed by swab and one with a suspected CT-scan with the negative swab.

The nasopharyngeal swab test was performed on all clinically or radiologically suspected patients. Therefore, even if it is not possible to assess the incidence and prevalence of SARS-CoV2 infection in our cohort, the rate of symptomatic and severe COVID-19 appears to be reliable data.

Indeed, in 5 weeks of multilevel measure, only six out of the 325 (1.8%) evaluated patients with lung cancer tested positive for SARS-CoV-2 infection, and only 1 patient (0.3%) required oxygen support due to severe COVID-19 and no deaths occurred.

The incidence rate was higher compared to the general population since the cumulative incidence of SARS-CoV2 infection in the Lombardy region is about 0.5% (457.94 over 100,000 inhabitants). However, it is necessary to consider that Italian National Health Service does not recommend performing swab tests in asymptomatic cases, while three out of six of our positive patients underwent swab tests after a suspect CT scan, which was regularly planned for cancer evaluation. Those patients presented with no or minimal symptoms and would not have been tested for COVID-19 in the absence of a suspicious CT scan. Another limitation in interpreting our results may be related to the different timing of adoption of the containment measures; during a pandemic, management has been gradually implemented according to both Institute internal actions and national/international indications. As a consequence, the results of our measures may dependent on time, and a more extensive observation will be likely to observe even better outcomes in terms of cancer care management.

## Conclusion

In a warning scenario for medical oncologists and their patients with lung cancer, these data from our Institute suggest that a resources prioritization is mandatory, confirming that proactive and multilevel management is essential to date in clinical practice to minimize the COVID-19 spread in the hospital setting, preventing patients and stakeholders from the potential contagious. For patients with lung cancer, characterized by the high potential risk of COVID-19, these management approaches should be implemented by a robust and structured testing analysis, based on approved government directions, to identify in advance patients positive for SARS-Cov-2.

Our mission is to guarantee that no patients should be left behind in the path of supportive or curative treatment and to do this. A joint effort is needed during this pandemic.

## Data Availability Statement

The raw data supporting the conclusions of this article will be made available by the authors, without undue reservation.

## Ethics Statement

Ethical review and approval was not required in accordance with the local legislation and institutional requirements. Written informed consent to participate in research study was provided by the patients. Written informed consent was obtained from all the evaluated patients for the publication of data.

## Author Contributions

FM and AP designed the research study. IA, SM, LG, GS, VS, and ED were involved in data collection. FM, IA, and AP were involved in data analysis. IA, SM, LG, GS, VS, and AP were responsible for the writing of the manuscript. All the authors contributed to revised and reviewed the manuscript and all the data reported.

## Conflict of Interest

The authors declare that the research was conducted in the absence of any commercial or financial relationships that could be construed as a potential conflict of interest.

## References

[1] Data from: Coronavirus COVID-19 Global Cases by the Center for Systems Science and Engineering (CSSE) at Johns Hopkins University (JHU) ArcGIS. Johns Hopkins CSSE. Available online at: https://coronavirus.jhu.edu/map.html (accessed April 4, 2020).

[2] Onder G Rezza G Brusaferro S. Case-fatality rate and characteristics of patients dying in relation to COVID-19 in Italy. JAMA. (2020). 10.1001/jama.2020.4683 32203977

[3] Liang W Guan W Chen R Wang W Li J Xu K. Cancer patients in SARS-CoV-2 infection: a nationwide analysis in China. Lancet Oncol. (2020) 21:335–7. 10.1016/S1470-2045(20)30096-6 32066541 PMC7159000

[4] Novel Coronavirus Pneumonia Emergency Response Epidemiology Team The epidemiological characteristics of an outbreak of 2019 novel coronavirus diseases (COVID-19)—China. Zhonghua Liu Xing Bing Xue Za Zhi. (2020). 41:145–51. 10.3760/cma.j.issn.0254-6450.2020.02.003 32064853

[5] Yu J Ouyang W Chua MLK Xie C SARS-CoV-2 trasmission in patients with cancer at a tertiary care hospital in Wuhan, China. JAMA Oncol. (2020) e200980. 10.1001/jamaoncol.2020.0980 PMC7097836 32211820

[6] Passaro A Peters S Mok T Attili A Mitsudomi T de Marinis F. Testing for COVID-19 in lung cancer patients. Ann Oncol. (2020). 10.1016/j.annonc.2020.04.002 32278879 PMC7144604

[7] Guan WJ Ni ZY Hu Y Liang WH Ou CQ He JX Clinical characteristics of coronavirus disease 2019 in China. N Engl J Med. (2020). 10.1056/NEJMoa2002032 PMC7092819 32109013

[8] Zhang L Zhu F Xie L Wang C Wang J Chen R. Clinical characteristics of COVID-19-infected cancer patients: a retrospective case study in three hospitals within Wuhan, China. Ann Oncol. (2020). 10.1016/j.annonc.2020.03.296 32224151 PMC7270947

[9] Lambertini M Toss A Passaro A Criscitiello C Cremolini C Cardone C Cancer care during the spread of coronavirus disease 2019 (COVID-19) in Italy: young oncologist's perspective. ESMO Open. (2020) 5:e000759 10.1136/esmoopen-2020-000759 32229501 PMC7174009

[10] Ueda M Martins R Hendrie PC McDonnell T Crews JR Wong TL. Managing cancer care during the COVID-19 pandemic: agility and collaboration toward a common goal. J Natl Compr Canc Netw. (2020) 1–4. 10.6004/jnccn.2020.7560 32197238

[11] European Society of Medical Oncology, (2020). Available online at: https://www.esmo.org/newsroom/covid-19-and-cancer/supporting-oncology-professionals (accessed April 5, 2020).

[12] Banna G Curioni-Fontecedro A Friedlaender A Addeo A. How we teat patients with lung cancer during the SARS-CoV-2 pandemic: primum non nocere. ESMO Open. (2020) 5:e000765. 10.1136/esmoopen-2020-000765 32245904 PMC7211064

[13] Hanna TP Evans GA Booth CM. Cancer, COVID-19 and the precautionary principle: prioritizing treatment during a globalpandemic. Nat Rev Clin Oncol. (2020). 10.1038/s41571-020-0362-6 32242095 PMC7117554

[14] Associazione Italiana Oncologia Medica (2020). Available online at: https://www.aiom.it/rischio-infettivo-da-coronavirus-covid-19-indicazioni-aiom-comu-cipomo-per-loncologia/ (accessed April 5, 2020).

